# Quantitative Lipidomic Analysis of Takotsubo Syndrome Patients' Serum

**DOI:** 10.3389/fcvm.2022.797154

**Published:** 2022-04-19

**Authors:** Srikanth Karnati, Gulcan Guntas, Ranjithkumar Rajendran, Sergey Shityakov, Marcus Höring, Gerhard Liebisch, Djuro Kosanovic, Süleyman Ergün, Michiaki Nagai, Carola Y. Förster

**Affiliations:** ^1^University of Würzburg, Institute of Anatomy and Cell Biology, Würzburg, Germany; ^2^Department of Biochemistry, Medical Faculty, Atilim University, Ankara, Turkey; ^3^Experimental Neurology, Department of Neurology, Justus Liebig University, Giessen, Germany; ^4^Infochemistry Scientific Center, Laboratory of Chemoinformatics, ITMO University, Saint-Petersburg, Russia; ^5^Institute of Clinical Chemistry and Laboratory Medicine, University Hospital of Regensburg, Regensburg, Germany; ^6^Department of Pulmonology, I. M. Sechenov First Moscow State Medical University (Sechenov University), Moscow, Russia; ^7^Hiroshima City Asa Hospital, Department of Cardiology, Hiroshima, Japan; ^8^University of Würzburg, Department of Anaesthesiology, Intensive Care, Emergency and Pain Medicine, Würzburg, Germany

**Keywords:** TTS, inflammation, lipids, TNF-α, IL6, PIK3R1, NF-kappa-B, phosphatidylinositol

## Abstract

Takotsubo syndrome (TTS), also known as the transient left ventricular apical ballooning syndrome, is in contemporary times known as novel acute cardiac syndrome. It is characterized by transient left ventricular apical akinesis and hyperkinesis of the basal left ventricular portions. Although the precise etiology of TTS is unknown, events like the sudden release of stress hormones, such as the catecholamines and the increased inflammatory status might be plausible causes leading to the cardiovascular pathologies. Recent studies have highlighted that an imbalance in lipid accumulation might promote a deviant immune response as observed in TTS. However, there is no information on comprehensive profiling of serum lipids of TTS patients. Therefore, we investigated a detailed quantitative lipid analysis of TTS patients using ES-MSI. Our results showed significant differences in the majority of lipid species composition in the TTS patients compared to the control group. Furthermore, the computational analyses presented was able to link the altered lipids to the pro-inflammatory cytokines and disseminate possible mechanistic pathways involving TNFα and IL-6. Taken together, our study provides an extensive quantitative lipidome of TTS patients, which may provide a valuable Pre-diagnostic tool. This would facilitate the elucidation of the underlying mechanisms of the disease and to prevent the development of TTS in the future.

## Introduction

Takotsubo syndrome (TTS), also known as broken heart syndrome, stress induced cardiomyopathy, and Takotsubo cardiomyopathy, is an acute cardiac syndrome with rapid onset of chest pain and dyspnea (1–6). TTS is often triggered by physical and emotional stress and is characterized by a transient and reversible severe left ventricular dysfunction which typically recovers spontaneously within hours to weeks (7). The prevalence of TTS has been reported to be ~2–3% of all patients with clinical appearance of acute coronary syndrome (ACS) (8). TTS is a remarkably like ACS with almost the same clinical presentations and ST elevations; therefore, differential diagnosis is critically important in the emergency department.

Despite the pathogenesis of TTS not being fully understood, several pathophysiological mechanisms have been suggested. These could include the following: myocardial ischemia, left ventricular outlet obstruction, increased circulating and myocardial catecholamine levels with myocardial toxicity, endothelial dysfunction, epinephrine-induced switch in signal trafficking, and autonomics nervous system dysfunction with cardiac sympathetic activation including over stimulation of beta receptors (4, 7, 9, 10).

The predominant pattern of TTS is widespread dyskinesia in the apical segments and hyperkinesia in the basal segment of left ventricle (LV) with apical ballooning (9, 11). It is suggested that local differences in adrenergic receptors may be the explanation of this involvement in the LV (11). Experimental studies have shown that the LV in canine has β2-adrenoceptors (β2-AR) that are expressed much more in the apical than in the basal segments (11, 12). Feola et al. also corroborated this hypothesis with a myocardial PET study showing decreased coronary flow reserve and impaired metabolism in the apical segments during the acute phase of TTS (13).

Several studies have shown that adrenergic overstimulation is strongly associated with the pathogenesis of TTS, but the mechanism remains unclear (7–9, 12, 14). The acute phase of TTS is characterized by supraphysiological levels of circulating and cardiac catecholamine (15) which operate as positive inotropic and chronotropic effects on the heart and regulate the myocardial lipid metabolism (7, 14, 16). The heart provides energy from the utilization of lipids. It uptakes lipid by protein transporters and secretes its own ApoB lipoproteins to excrete excess lipids. Excessive plasma catecholamine levels can lead to the disruption of the myocardial lipid metabolism in the heart (14).

Lipids have an important role in cellular energy storage, structure, and signaling. In addition to being a component of membranes, lipids play an essential role in the immune response by regulating signaling complexes in the cellular membrane (17). Recent studies have indicated that lipid metabolic disorders can cause various human diseases (17, 18). The human plasma lipid profile of TTS is so far poorly understood. A detailed lipid analysis of TTS patients could provide a valuable development to elucidate the underlying mechanisms of the disease.

In the current study, a total lipid profiling was performed including the measurement of 140 glycerophospholipids (GP), 44 sphingolipids (SL), 20 sterols (ST), and 58 glycerolipids (GL) in the control, acute TTS, and subacute TTS groups. To our knowledge, this study is the first to measure such an extensive quantitative lipidome analysis in TTS to date. The aim of our study was to investigate the underlying pathophysiological mechanism of TTS in relation to lipid metabolism and to relate it to the pro-inflammatory processes described to be prevalent in this syndrome.

## Materials and Methods

### Materials

All chemicals were purchased from Sigma-Aldrich (Deisenhofen, Germany) unless otherwise mentioned. Phospholipid standards were obtained from the Avanti Polar Lipids (Alabaster, AL, USA), standards of cholesterol and cholesteryl esters with purity >95% obtained from Sigma (Taufkirchen, Germany). High purity cholesterol-(25, 26, 26, 26, 27, 27, 27-d7) was purchased from Cambridge Isotope Laboratories (Andover, MA, USA). HPLC grade solvents methanol and chloroform were obtained from Merck (Darmstadt, Germany). Analytical grade ammonium acetate and acetyl chloride were obtained from Sigma-Aldrich (Buchs, Switzerland). All other reagents used were of high purity and analytical grade.

### Ethics Approval and Consent to Participate

The study protocol was approved by the Hiroshima City Asa Hospital Research Committee (01-3-3), Hiroshima, Japan and was conducted in accordance with the principles stated in the Declaration of Helsinki. All participants provided informed written consent. From July 2019 to September 2019, 9 patients with hospitalized TTS and 6 healthy controls were registered consecutively at Hiroshima City Asa Hospital. While all eligible subjects were included in this analysis, none of the TTS patients and healthy controls were died during hospitalization”. In the Kruskall Wallis test, there were no significant difference in the age (median age 83 vs. 80 yrs, *p* = 0.48) and female percentage (78 vs. 100%, *p* = 0.23) between the group with TTS and control.

### Human Patient Serum

All human blood from patients providing informed written consent was sampled using S-Monovette collection tubes (Sarstedt). We collected blood samples of 15 pseudonymized patients which were subsequently divided into 3 different groups: control (*n* = 6), patients with acute TTS (first 2 weeks after onset, *n* = 4), patients with subacute TTS (2–6 weeks after onset, *n* = 5). The healthy controls did not present with altered coronary arteries in the coronary angiography diagnostics, while minor cardiovascular or endocrinological diseases were accepted (e.g.,: arterial hypertension, diabetes mellitus). Patient's baseline demographic and vascular risk factors characteristics are summarized in Table 1. All patients were diagnosed according to the InterTAK Diagnostic Criteria (19) as was described below:

**Table 1:** Baseline demographic and vascular risk factors.

	**Acute TTS (*n* = 4)**	**Subacute TTS (*n* = 5)**	**Control (*n* = 6)**	***p*-value**
Age (yrs)	76	88	80	*p* = 0.21
Female (%)	50	100	100	*p* = 0.052
Smoking (%)	50	0	0	*p* = 0.052
Hypertension (%)	50	40	67	*p* = 0.69
Lipid disorder (%)	25	20	67	*p* = 0.25
Diabetes mellitus (%)	0	0	17	*p* = 0.47

*Data are presented as the median or as percentages. p-value comparison across three groups using an Kruskal-Wallis test for qualitative variables*.

For serum preparation, all the blood was drawn from subjects in using S-Monovette collection tubes (Sarstedt) and incubated at room temperature for 60 min. Then, the blood samples were centrifuged at 1,500 × g for 15 min, the serum was isolated, immediately frozen and stored at −80°C until the extraction. All the methods used in this study were performed in accordance with the relevant guidelines and regulations. Serum samples were quantified and analyzed individually.

### Lipid Extraction and Sample Preparation

**Figure 1:**
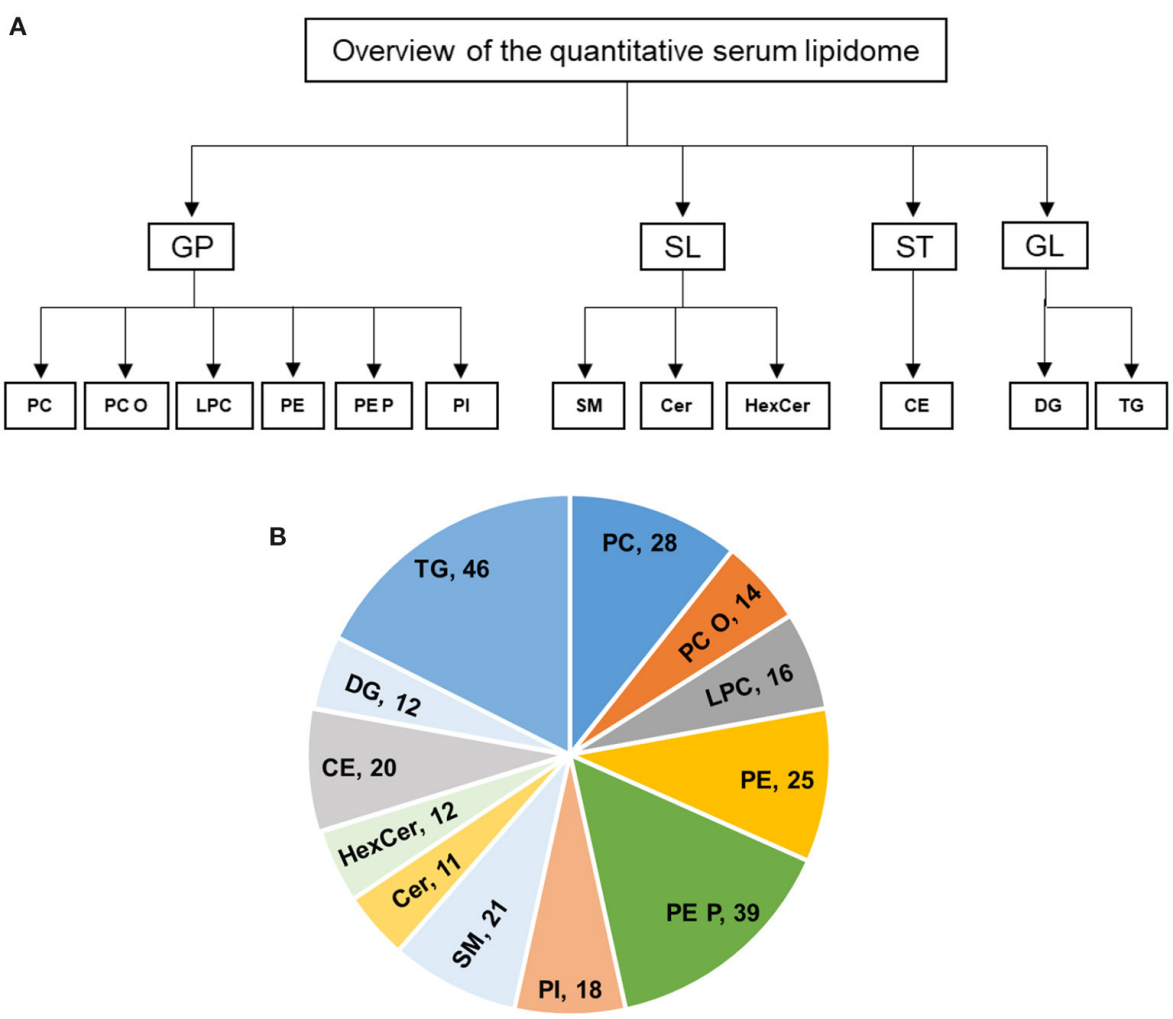
**(A,B)** Overview of the quantitative lipidomic analyses of study groups by mass spectrometry. The numbers represent number of lipid species quantified for lipid class.

Fresh snap-frozen serum samples were transported to University hospital Würzburg in dry ice. Serum samples were quantified and 5 μl of each subject were used for extraction, and lipids were extracted according to the procedure described by Bligh and Dyer (20). The following lipid species were added as internal standards: PC 14:0/14:0, PC 22:0/22:0, PE 14:0/14:0, PE 20:0/20:0 (di-phytanoyl)c, PS 14:0/14:0, PS 20:0/20:0 (di-phytanoyl), PI 17:0/17:0, LPC 13:0, LPC 19:0, LPE 13:0, Cer d18:1/14:0, Cer d18:1/17:0, D7-FC, CE 17:0, CE 22:0, TG 51:0, TG 57:0, DG 28:0 and DG 40:0. Chloroform phase was recovered by a pipetting robot (Tecan Genesis RSP 150) and vacuum dried. The residues were dissolved in either in 10 mM ammonium acetate in methanol/chloroform (3:1, v/v) (for low mass resolution tandem mass spectrometry) or chloroform/methanol/2-propanol (1:2:4 v/v/v) with 7.5 mM ammonium formate (for high resolution mass spectrometry). In the present study, we analyzed the following lipids: PC, PC O, LPC, PE, PE P, PI, SM, Cer, HexCer, CE, DG, and TG (Figure 1A).

### Mass Spectrometric Analysis of Lipids

The analysis of lipids was performed by direct flow injection analysis (FIA) using a triple quadrupole mass spectrometer (FIA-MS/MS; QQQ triple quadrupole) and a hybrid quadrupole-Orbitrap mass spectrometer (FIA-FTMS; high mass resolution).

FIA-MS/MS (QQQ) was performed in positive ion mode using the analytical setup and strategy described previously (21). A fragment ion of m/z 184 was used for lysophosphatidylcholine (LPC) (22). The following neutral losses were applied: Phosphatidylethanolamine (PE) 141, phosphatidylserine (PS) 185, phosphatidylglycerol (PG) 189 and phosphatidylinositol (PI) 277 (23). PE-based plasmalogens (PE P) were analyzed according to the principles described by Zemski-Berry (24). Sphingosine based ceramides (Cer) and hexosylceramides (HexCer) were analyzed using a fragment ion of m/z 264 (25). Quantification was achieved by calibration lines generated by addition of naturally occurring lipid species to the respective sample matrix. Calibration lines were generated for the following naturally occurring species: PC 34:1, 36:2, 38:4, 40:0 and PC O-16:0/20:4; SM 18:1;O2/16:0, 18:1, 18:0; LPC 16:0, 18:1, 18:0; PE 34:1, 36:2, 38:4, 40:6 and PE P-16:0/20:4; PS 34:1, 36:2, 38:4, 40:6; Cer 18:1;O2/16:0, 18:0, 20:0, 24:1, 24:0; FC, CE 16:0, 18:2, 18:1, 18:0.

The Fourier Transform Mass Spectrometry (FIA-FTMS) setup is described in detail in Höring et al. (26). Triglycerides (TG), diglycerides (DG) and cholesteryl ester (CE) were recorded in positive ion mode FTMS in range m/z 500–1,000 for 1 min with a maximum injection time (IT) of 200 ms, an automated gain control (AGC) of 1^*^106, three microscans and a target resolution of 140,000 (at m/z 200). Phosphatidylcholine (PC), sphingomyelin (SM) were measured in range m/z 520–960. Multiplexed acquisition (MSX) was used for, the [M+NH4]+ of free cholesterol (FC) (m/z 404.39) and D7-cholesterol (m/z 411.43) 0.5 min acquisition time, with a normalized collision energy of 10%, an IT of 100 ms, AGC of 1^*^105, isolation window of 1 Da, and a target resolution of 140,000 (27). Data processing details were described in Höring et al. (26) using the ALEX software (28) which includes peak assignment and intensity picking. The extracted data were exported to Microsoft Excel 2016 and further processed by self-programmed Macros. FIA-FTMS quantification was performed by multiplication of the spiked IS amount with analyte-to-IS ratio.

Lipid species were annotated according to the latest proposal for shorthand notation of lipid structures that are derived from mass spectrometry (29). For QQQ glycerophospholipid species annotation was based on the assumption of even numbered carbon chains only. SM species annotation is based on the assumption that a sphingoid base with two hydroxyl groups is present. Final quantities of lipid species and total lipid (sum of analysed lipid species) were calculated and expressed in nanomoles per milliliter of serum samples.

### Statistics

All data are expressed as mean ± standard deviation (SD) from control (*n* = 6), acute TTS (*n* = 4), and subacute TTS (*n* = 5) groups. Two-way analysis of variance (ANOVA) was calculated using GraphPad Prism 9.1.0 (GraphPad Software, California, USA) and statistical comparisons between the groups were performed by Tukey's multiple comparisons Post-test using the same software. Graphs were prepared using the same GraphPad Prism 9.1.0 software. A *p*-value of 0.05 or lower was considered as significant. Significance is indicated as ^*^ *P* ≤ 0.05, ^**^ *P* ≤ 0.01, ^***^ *P* ≤ 0.001, ^****^ *P* ≤ 0.0001.

### Computational Methods

The Cytoscape v.3.7.1 software was used to search for the specific pathways and networks of analyzed lipids in humans (30). In particular, we implemented the search mode by lipid IDs. These were taken from the ChEBI (Chemical Entities of Biological Interest) server as a freely available dictionary of molecular entities focused on “small” chemical compounds various databases. Those including IMEx, UntAct, MINT, UniProt, BioGrid, iRefIndex, tfact2gene, bhf-ucl, HPIDd, mentha, EBI-GOA-nonIntAct, Reactome-FIs, MPIDB, MatrixBD, and MBIInfo and were screened to find relevant protein-lipid pathways associated with the proinflammatory cytokines, such as IL-6 and TNF-alpha (31).

## Results

### Overview of the Quantitative Serum Lipidome

In this study, the serum lipid profiles of 262 individual lipid species and cholesterol were determined with lipidomics from serum samples in controls (*n* = 6), acute TTS (*n* = 4), and subacute TTS (*n* = 5). The 262 individual lipid species consists of 140 glycerophospholipids (28 PC, 14 PC O, 16 LPC, 25 PE, 39 PE P, and 18 PI), 44 sphingolipids (21 SM, 11 Cer, and 12 HexCer), 20 sterols (20 CE), and 58 glycerolipids (12 DG and 46 TG). An overview of the total lipid analysis of the study groups is shown in Figures 1A,B.

### Individual Lipid Species Analysis Between Three Study Groups

The current study evaluated the individual lipid alterations in acute and subacute TTS patients and controls. Statistically significant differences in lipid compositions in the TTS patients were observed compared to the control group. These differences of individual lipid species were shown to be generally lower in acute TTS as compared to the control and subacute groups.

### Glycerophospholipids Species

**Figure 2:**
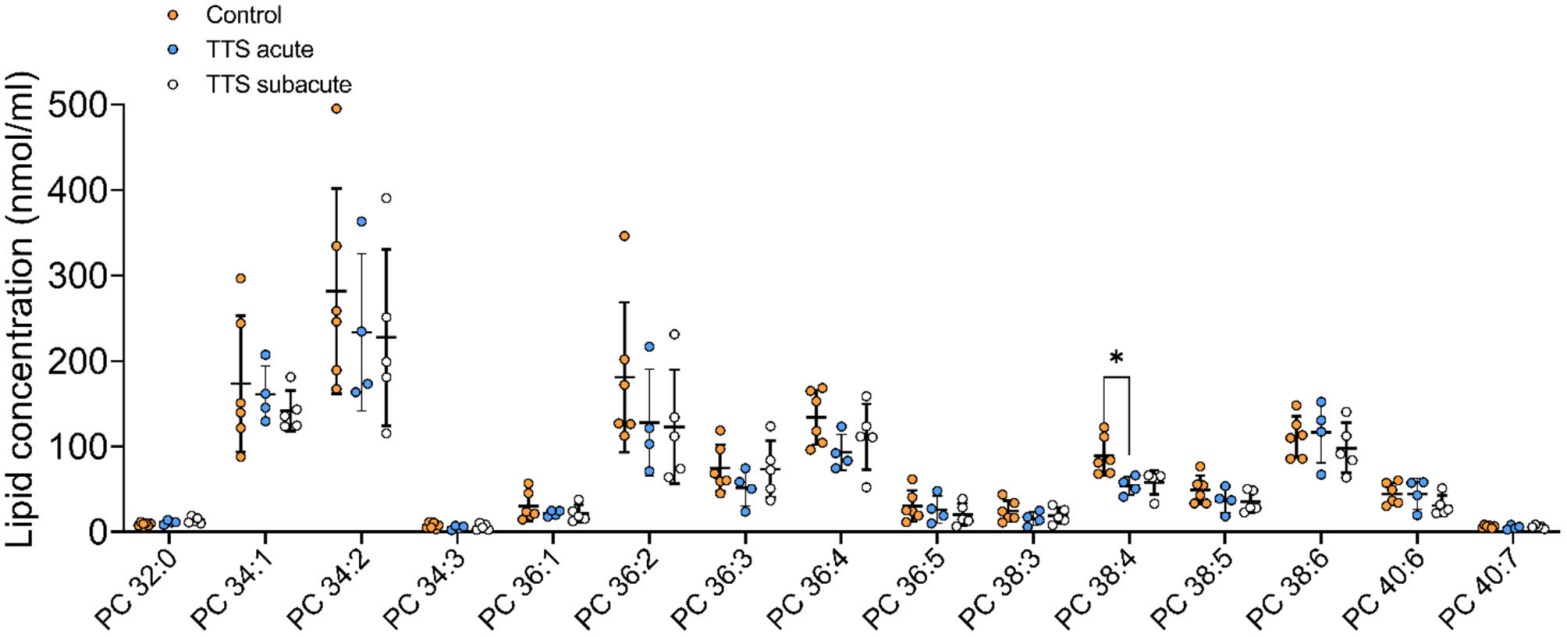
Composition of individual phosphatidylcholine (PC) lipid species in the acute TTS, subacute TTS, and control groups. Values are represented as nmol/ml of serum. Values are mean ± SD, *P*-value: *P < 0.05. Where significance is not mentioned, values are considered as being not significant. PC; Phosphatidylcholine, TTS, Takotsubo syndrome.

**Phosphatidylcholine (PC)**. In total, 28 PC species with different chain length and degree of unsaturation were analyzed in the acute TTS, subacute TTS, and control groups. Their compositions (15 PC species) are represented in Figure 2.

The polyunsaturated specie PC 38:4 was slightly decreased in the acute TTS group, as compared to the control group (*P* < 0.05) (Figure 2).

**Figure 3:**
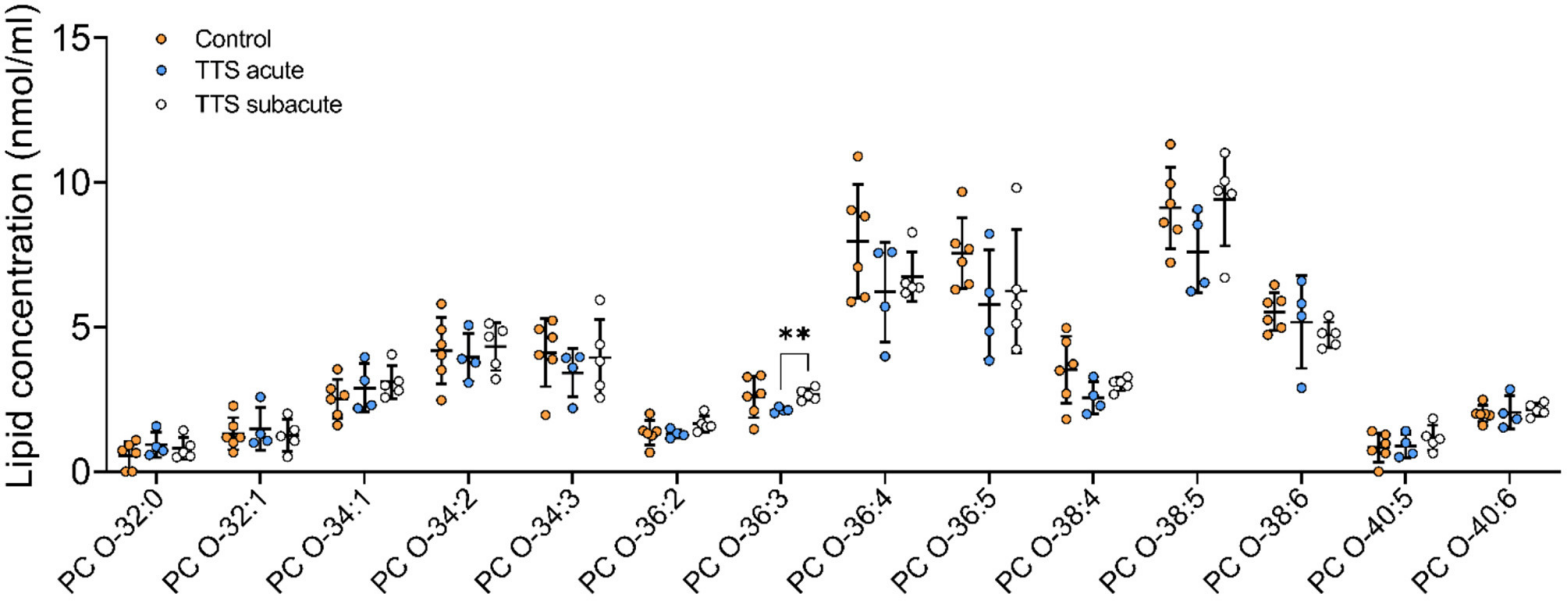
Composition of individual PC O lipid species in the acute TTS, subacute TTS, and control groups. Values are represented as nmol/ml. Values are mean ± SD, *P*-value: **P < 0.01. Where significance is not mentioned, values are considered as being not significant. PC O, Ether-phosphatidylcholine; TTS, Takotsubo syndrome.

**Ether-phosphatidylcholine (PC O)**. There was a statistical difference between the acute TTS and the subacute TTS in PC O-36:3 (*P* < 0.01), no significant difference was observed between all three groups in other PC O species (*P* > 0.05) (Figure 3).

**Figure 4:**
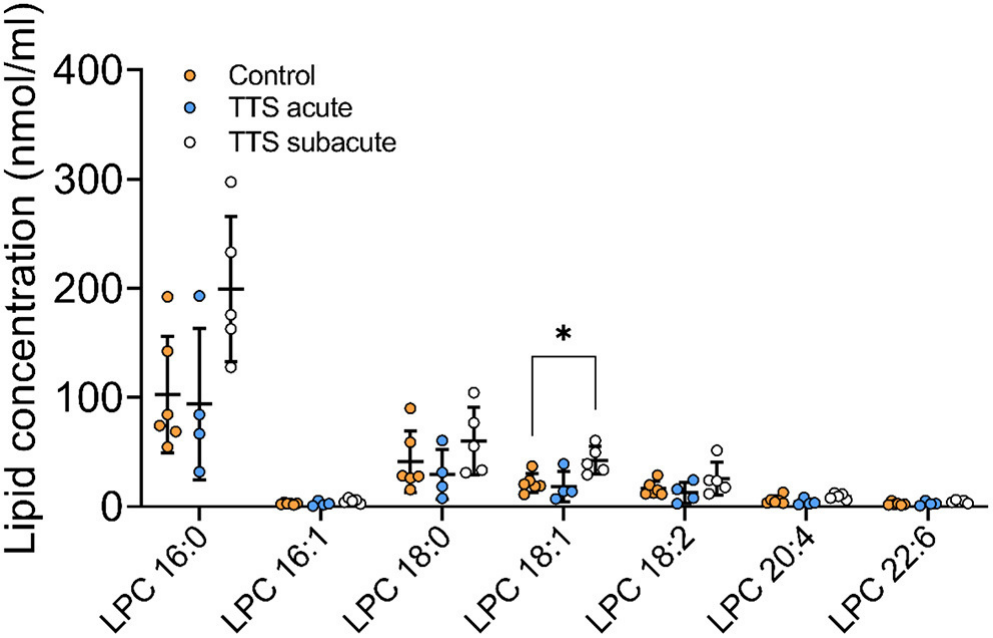
Composition of individual LPC lipid species in the acute TTS, subacute TTS, and control groups. Values are represented as nmol/ml. Values are mean ± SD, *P*-value: *P < 0.05. Where significance is not mentioned, values are considered as being not significant. LPC, Lysophosphatidylcholine; TTS, Takotsubo syndrome.

**Lysophosphatidylcholine (LPC)**. Of the 16 total LPC species analyzed, 7 LPC species were showed in the Figure 4, only one was significantly different, and the remaining LPC species did not reveal any differences (Figure 4). The LPC 18:1 showed slightly increased levels in the subacute TTS patients than those in the controls (*P* < 0.05).

**Figure 5:**
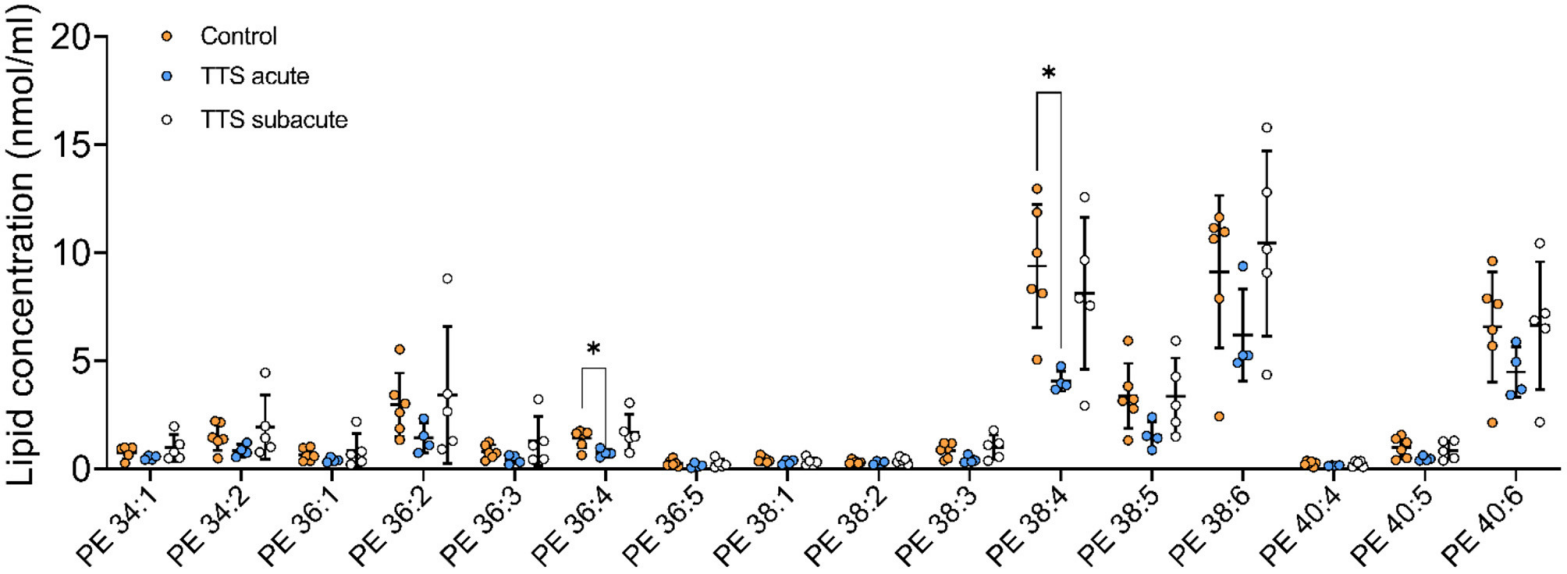
Composition of individual PE lipid species in the acute TTS, subacute TTS, and control groups. Values are represented as nmol/ml. Values are mean ± SD, *P*-value: *P < 0.05. Where significance is not mentioned, values are considered as being not significant. PE, Phosphatidylethanolamine; TTS, Takotsubo syndrome.

**Phosphatidylethanolamine (PE)**. We analyzed 25 individual lipid species of PE and 16 species compositions in all three groups is depicted in Figure 5. The most notably increased PE species were the long chain polyunsaturated species included PE 38:4 and PE 38:6. PE 38:4 and PE 36:4 significantly reduced in the acute TTS group in comparison to the controls (*P* < 0.05, *P* < 0.05, respectively). Remaining PE species did not represent any statistical difference in all groups.

**Figure 6:**
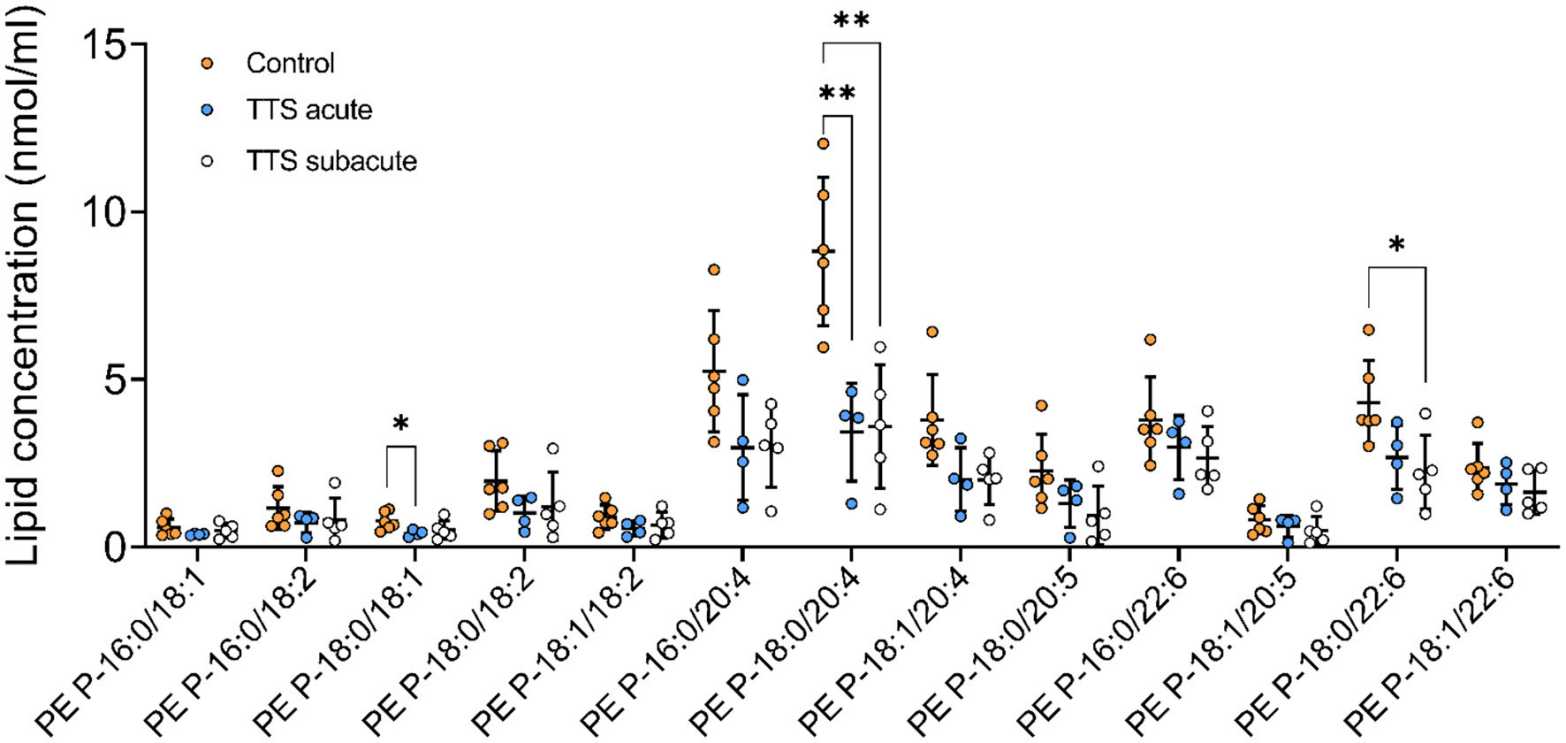
Composition of individual PE P lipid species in the acute TTS, subacute TTS, and control groups. Values are represented as nmol/ml. Values are mean ± SD, *P*-value: **P < 0.01, *P < 0.05. Where significance is not mentioned, values are considered as being not significant. PE P, PE based plasmalogen; TTS, Takotsubo syndrome.

**Phosphatidylethanolamine based plasmalogens (PE P)**. The individual composition of 39 species of PE P were analyzed and 13 species were depicted in Figure 6. The PE P-18:0/18:1 and PE P-18:0/20:4 had a significantly decreased serum levels in the acute TTS patients compared to the control group. The PE P-18:0/22:6 showed a significant decreased in the subacute TTS patients compared to the control group.

**Phosphatidylinositol (PI)**. We analyzed 18 individual lipid species of PI and 12 species composition is displayed in Supplementary Figure 1. PI lipid species did not indicate any statistical differences in all three groups.

### Sphingolipid Species

**Sphingomyelin (SM)**. In total, 21 different SM species were analyzed and 13 species in the three groups are shown in Supplementary Figure 2. There was no significant difference in SM species in all three groups.

**Figure 7:**
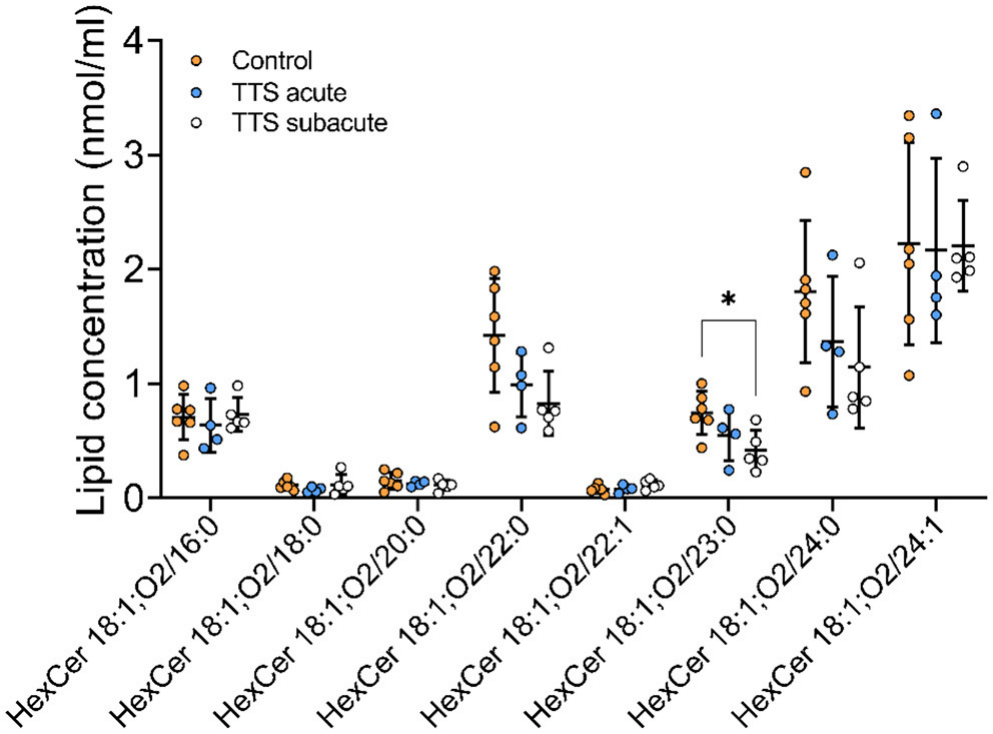
Composition of individual HexCer lipid species in the acute TTS, subacute TTS, and control groups. Values are represented as nmol/ml. Values are mean ± SD, *P*-value: *P < 0.05. Where significance is not mentioned, values are considered as being not significant. HexCer, Hexosylceramide; TTS, Takotsubo syndrome.

**Ceramide (Cer) and Hexosylceramide (HexCer)**. A total of 11 Cer and 12 HexCer species were analyzed, and their compositions are shown in Supplementary Figure 3 and Figure 7, respectively. There was no significant difference in Cer species between all three groups.

HexCer 18:1; O2/23:0 was significant reduced in subacute TTS group when compared to the control group. Remaining species of HexCer did not show any statistically differences in the all three groups (Figure 7).

### Sterol Species

**Cholesteryl esters (CE)**. We analyzed 20 CE species along with their compositions (Supplementary Figure 4). There was not observed any statistical difference in the CE species.

### Glycerolipid Species

**Figure 8:**
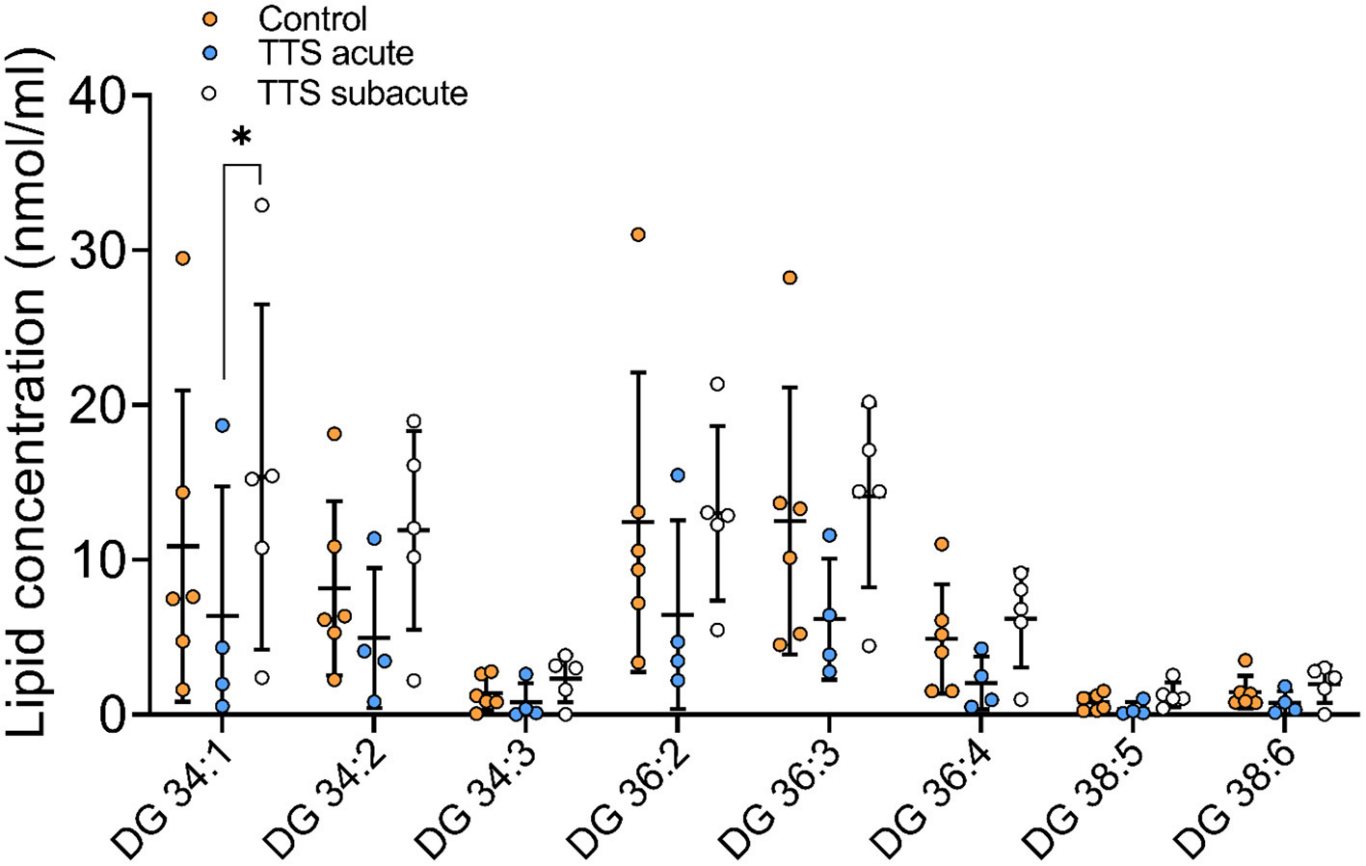
Composition of individual DG lipid species in the acute TTS, subacute TTS, and control groups. Values are represented as nmol/ml. Values are mean ± SD, *P*-value: *P < 0.05. Where significance is not mentioned, values are considered as being not significant. DG, Diacylglycerol; TTS, Takotsubo syndrome.

**Diacylglycerol (DG) and Triacylglycerol (TG) species**. Individual 12 DG were analyzed, and 8 species were displayed in Figures 8 and 46 TG species were analyzed and 14 species compositions are depicted in Supplementary Figure 5.

DG 34:1 showed a slight decrease in the acute TTS group as compared to the subacute group. Interestingly, there was no significant difference between the acute TTS and control groups in DG species. There was not observed any statistical differences in the TG species between the all groups.

### Computational Analysis

**Figure 9:**
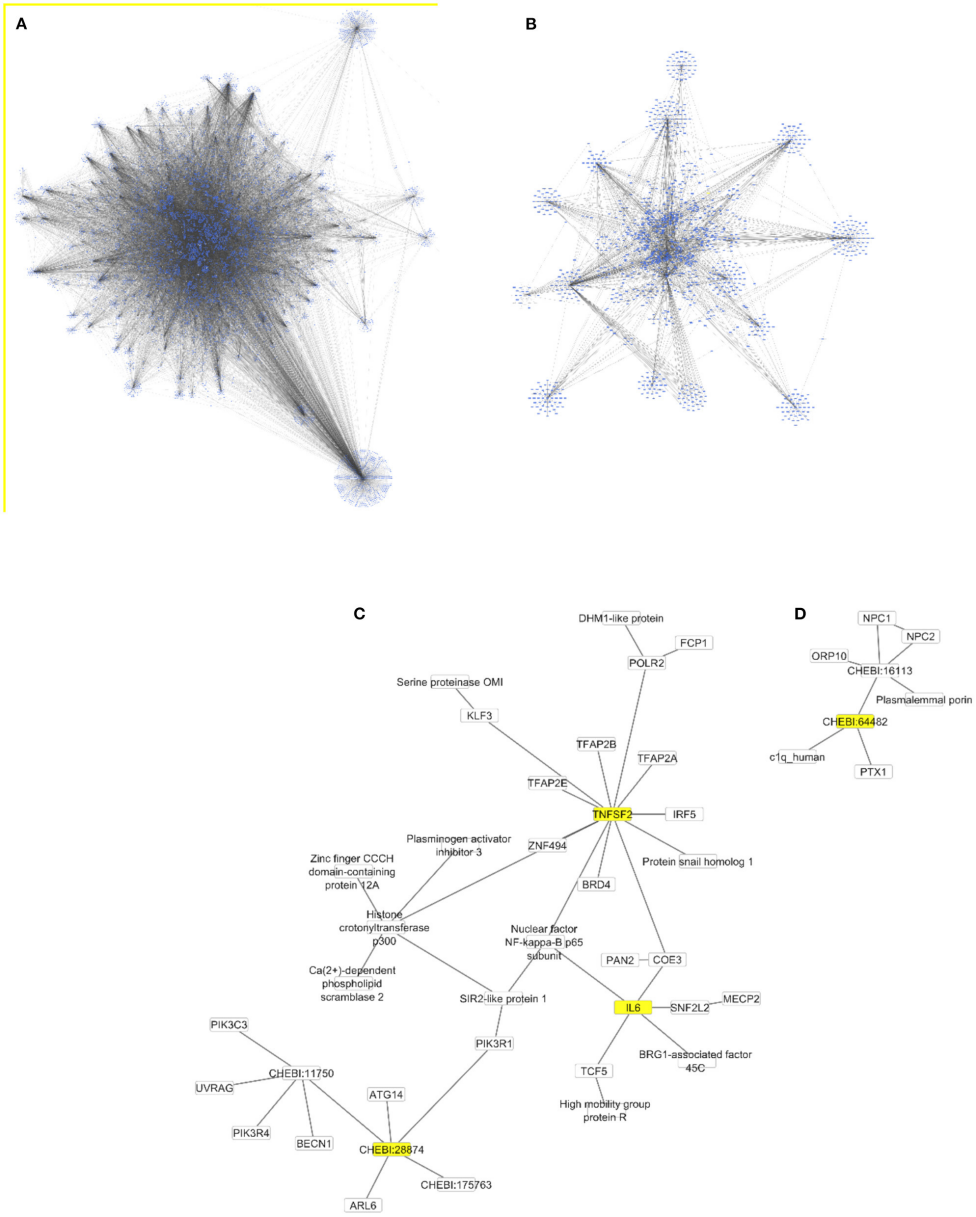
Protein-lipid networks linked to the proinflammatory cytokines [IL6 and TNF-α (TNFSF2)] as complete **(A)**, using the first directed **(B)** and undirected **(C,D)** neighbors to phosphatidylinositol (CHEBI:28874) and phosphatidylcholine (CHEBI:64482) as nodes colored in yellow. All networks are displayed using the yFiles organic layout.

The Cytoscape algorithm was able to reconstitute a detailed molecular pathway linked to IL-6 and TNF-α for the two lipids (phosphatidylinositol and phosphatidylcholine) used in the study. Both lipids were present in the complete human protein-lipid network comprising 14,277 nodes and 37,778 edges (Figure 9A). This network was processed to reduce the number of interacting nodes (1,272) and edges (2,545) by using the first directed (incoming and outgoing) neighbors of analyzed nodes (Figure 9B). Finally, the two separate networks were produced for each lipid using the first directed and undirected neighbors of analyzed nodes (Figures 9C,D). In particular, a detailed molecular pathway was only predicted for phosphatidylinositol indirectly linked to the proinflammatory cytokines through NF-kappa-B, such as phosphatidylinositol–PIK3R1–SIR2 like protein1–NF-kappa-B p65 subunit–IL6/TNF-α (Figure 9C).

## Discussion

In the current study, we have analyzed serum lipidomic profiling of TTS patients and controls. The data obtained from these analyses demonstrate for the first time a distinct composition and quantity of serum lipids of TTS patients as compared to the controls. We analyzed 262 lipid species which consist of GP, SL, ST and GL species. We observed lower levels of individual lipid species other than lysoglycerophospholipids (LGPLs) in acute TTS compared to the control and subacute TTS groups. The LPCs were significantly elevated in the subacute TTS patients compared to the acute TTS and control group.

TTS is an acute cardiac syndrome with akinesia of the LV and apical ballooning (9, 12, 32). Although adrenergic overstimulation is associated with initiating TTS, the underlying pathophysiological mechanisms are not clearly understood. To better understand the disease mechanism of TTS, identification of molecular biomarkers has great importance. It has been suggested that akinetic/dyskinetic segments of the heart may decrease the lipid and glucose uptake and lead to metabolic perturbations in acute TTS (14, 33). Lipids are the essential structural components of cardiomyocyte plasma and organelle membranes and have critical roles in cellular functions, including energy storage and signal transduction (34). The metabolism of lipid is indicated in several human diseases such as CVD, respiratory disease, diabetes, and Alzheimer's disease (18, 35, 36). The normal heart regulates uptake and oxidation of fatty acids to sustain membrane biosynthesis and lipid signaling (37, 38). Since fats are the primary sources for cardiac energy, lipids have a critical role in the heart. Lipidomic profiling enables us to quantify the composition of individual lipids and molecular species that reveal metabolic variation in the structure. Although lipid alterations have been demonstrated in a limited number of studies in TTS (14, 39–41), the potential role of lipid metabolism in the pathology of TTS remains unclear. Therefore, lipidomics profiling could provide valuable information regarding the pathophysiology of TTS disease. Our work is the first comprehensive investigation into lipidome analysis using electrospray ionization-tandem mass spectrometry (ESI-MS/MS) in TTS patients.

The GPs are the main molecules for the backbone of cellular membranes and are involved in cellular signal transduction (42, 43). We analyzed composition ratio of 140 GPs and found that 8 GPs species were present significantly altered in serum from patients with TTS, including 1 PC, 1 PC O, 1 LPC, 2 PE, and 3 PE P. The long chain and polyunsaturated PC 38:4 was slightly decreased in the acute TTS group when compared to the control group (Figure 2). Our results were compatible with the research of Shao et al. (14), which is one of the very limited published studies on TTS and GPs. In this research, they created a stress induced cardiomyopathy (SIC) model with isoprenaline injection in mice. Consistent with our results, they found low PC levels in the plasma of the mice and SIC patients. In addition, they detected severe lipid accumulation and downregulations of ApoB gene expression in the myocardium in ISO-treated mice and SIC patients which may explain these results. It is suggested that catecholamine-induced akinetic/dyskinetic segments of the heart and impaired coronary blood flow reduced lipid uptake capacity during the acute phase of TTS (11, 13, 14, 41). It has been speculated that the increased myocardial lipid burden impairs efficient cardiac lipid export by ApoB, and therefore lower circulating lipid levels are seen in patients with TTS (14, 41). A further lipidomic study performed in the serum of dilated cardiomyopathy patients indicated significantly lower levels of TGs and PCs in line with our study (44). We detected statistically decrease in the PC 38:4, whereas Sysi-Aho and colleagues (44) found significant decrease in PC 38:2/38:5. These results showed that the number and species of altering GP species differs among different type of the disease. The functional importance and the role of altered differences and its mechanism should be clarified in further studies.

Lysoglycerophospholipids (LGPLs) are lacking one FA moiety in their structures. The main Lyso-GPLs are lysophosphatidylcholine (LPC), lysophosphatidic acid (LPA), lysophosphatidylinositol (LPI), and lysophosphatidylethanolamine (LPE) (18, 45). LGPLs have a similar effect to inflammatory lipids that modulates proliferation and apoptosis of endothelial cells in several diseases (18, 46, 47). In our study, we found that LPC 18:1 was significantly increased in the subacute TTS serum in comparison to the controls (Figure 4). This result showed the opposite of our PC results. The LPC is derived from PC, respectively in lipoproteins or from cell membranes *via* phospholipase A2 (18, 42). It is reported that LPC is elevated in inflammation-associated diseases and exerts its inflammatory effects *via* NF-kB, T-lymphocytes, monocytes, and neutrophils (47). Inflammation has been implicated as one of the various mechanisms involved in the pathogenesis of TTS (48). Active inflammation was displayed in human Post-mortem hearts and experimental models of TTS (9, 48). In our study, we found significantly higher levels of LPC in TTS patients that provide supportive evidence of inflammatory process in TTS.

Lipid metabolism is essential for maintenance of normal function and structure of the heart (49). Indeed, the importance of cardiac lipid metabolism has been further emphasized by the recent publication in which we provide translational evidence that cardiac glycosphingolipids are required to maintain β-adrenergic signalling and contractile capacity in cardiomyocytes and to preserve normal heart function (50). It was found that LPC is important for normal function. LPC is a major phospholipid component in cell membranes accounting for 40% of total phospholipids in the heart tissue (34, 51, 52). Because of its amphiphilic property, LPC is readily incorporated into the lipid bilayers of the cell membrane, changing the physicochemical property of the cell membrane and thereby affecting the receptors, enzymes, and ion channels embedded in the membrane (53). LPC is also involved in the regulation of intracellular pH (54). Thus, we suggest that the disrupted lipid metabolism is very important risk for trigging TTS. However, we could not conclude the causality because the analysis in this study was cross-sectional manner.

Plasmalogens are a subclass of GPs that have a number of cellular functions, including neurochemical effects, cellular signaling, and functioning as scavengers in cellular membrane (55, 56). Deficiency of plasmalogens plays a role in cardiac failure, obesity, inflammation, and cancer (18, 56). In the current study, 3 PE-based plasmalogen (PE P) and 1 ether-phosphatidylcholine (PC O) significantly decreased in TTS in comparison to the controls. Membranes of myocardial cells, especially the sarcoplasmic reticulum, contain high amounts of PC and PE plasmalogens with arachidonic acid in the sn-2 position (56). In eicosanoid biosynthesis, plasmalogens act as a reservoir for PUFAs as they contain arachidonic (20:4), docosapentaenoic (22:5n−6) and docosahexanoic (DHA-22:6) acids in the sn-2 position (32, 34). In our study, we detected PE P-18:0/18:1, PE P-18:0/20:4, and PE P-18:0/22:6 species, which enriched with arachidonic acid, were significantly decreased in acute TTS and subacute TTS patients as compared to the control group. We think that the antioxidant properties of plasmalogens may have led to these results. It is reported that overstimulation of adrenoceptors leads to myocellular hypoxia in TTS (7, 14). In addition to decreased coronary flow in TTS, accumulation of lipid droplets in cardiomyocytes may lead to lipid oxidation in the myocardium. These metabolic alterations can cause oxidative changes in membrane phospholipids and disrupt the integrity of cell membranes (57). As expected under such oxidative conditions plasmalogens are decreased and functioning as an antioxidant.

As far as we know, the relationship between TTS and plasmalogen has not been evaluated. On the other hand, similar to our results, studies in coronary stenosis patients (56), and hypertensive patients showed decreased plasma ether lipids in serum. Pietilainen et al. reported that increased levels of LPCs, and decreased level in ether phospholipids suggested a link between plasmalogens and oxidative stress in human obesity (58). Based on the above results and the literature, our study supports the view that inflammation may play a role in TTS, which was also demonstrated by Wilson et al. (48).

We detected 44 sphingolipids (SLs) in serum samples and observed that 6 of them indicated statistical differences in TTS patients when compared to the controls. Perturbation in SLs plasma and tissue levels have been shown to increase the risk of cardiovascular disease (59, 60). It is reported that circulating Cer 18:1; O2/24:1, and Cer 18:1; O2/24:0 are associated with the risk of the incidence of major adverse cardiovascular events in healthy people (61). In the context of cardiomyopathy, increased ceramide levels or changes in ceramide compositions have been suggested to be toxic (62). The increased levels of SM and Cer were displayed in different type of cardiomyopathies (59, 62). To date, no sphingolipids studies have been associated with TTS in patient's serum. Our study performed from serum of TTS patients and the controls and in contrast to above studies, we detected a reduced level of HexCer in patients of TTS. First, these alterations could be due to the differences between different cardiac diseases. Second, research showed that impaired myocardial perfusion and overexpression of catecholamine can disturb myocardial lipid metabolism (14, 41). It was suggested that suppression of myocardial ApoB expression may prevent the export of the accumulated lipids and lead to a reduction of circulating lipids. More lipidomic studies are needed to understand the roles of sphingolipids in TTS.

We also analyzed 78 types of CE, DG, and TG species. Neutral lipids characterize a group of hydrophobic molecules and include TGs, DGs, cholesterol and its esters in mammals (18, 63). CEs, TGs and DGs are thought to be involved in several diseases including cardiovascular diseases, ischemic stroke, hypertension and dyslipidemia (18). In the current study, the extended chain and monounsaturated DG lipid specie was remarkably reduced in the acute TTS patients in comparison to the subacute TTS patients. Long-chain fatty acid (LCFA) is the main sources of cardiomyocytes. TGs are hydrolyzed by adipose triglyceride lipase and released LCFAs which are oxidized in mitochondria to produce ATPs for cardiac energy (64). LCFAs are released on demand and delivered to the heart *via* the circulation and paracrine and used as energy substrates (64). In normal circumstances, the stress-induced increase in cardiac energy demand can be compensated from hydrolyzing TGs. In contrast, in TTS patients, excessive catecholamine-mediated myocardial segmental akinesia reduces lipid uptake and leads to lipid accumulation in the heart (9, 14, 64). Further studies are required to investigate the meaning of alterations in the composition of these individual lipid species.

Our study provides a comprehensive quantitative lipidome analysis which may play an important role in the pathogenesis and management of TTS. Moreover, the reported use of computational methods allowed for a reconstitution of a detailed molecular pathway linking IL-6 and TNF-α for the two lipids (phosphatidylinositol and phosphatidylcholine). Besides lipidomic profiling in TTS, uncovering an intriguing diversity of targetable mechanisms can be exploited to prevent primary or recurrent TTS in the future. Inflammation and lipid signaling are intertwined modulators of homeostasis and immunity. Emerging studies indicate that in addition to the extensively studied eicosanoids and inositol phospholipids, many other lipid species act positively and negatively regulate inflammatory responses (65). Our computational analyses of the lipidomic profile in TTS points to a molecular pathway linked to IL-6 and TNF-α, the pro-inflammatory cytokines characteristically elevated in TTS patients (15) for the two lipids (phosphatidylinositol and phosphatidylcholine). This mechanism largely depends on the phosphatidylinositol 3-kinase regulatory subunit alpha (PIK3R1), which was previously observed mediating TNF-induced NF-kappa-B activation (66). In turn, IL-6 could induce phosphatidylinositol 3-kinase and nitric oxide-dependent protection and preserves mitochondrial function in cardiomyocytes (67). On the other hand, the long-term IL6 signaling or an over-production of IL6R protein could potentially lead to cardiovascular diseases followed by heart failure (68).

Takotsubo cardiomyopathy has already cardiac biomarkers, such as the NT-proBNP (N-terminal B-type natriuretic peptide)/myoglobin and NT-proBNP/troponin T ratios for the diagnosis of acute coronary syndromes and stress-induced cardiomyopathy (61). Based on the computational analysis of the TTS lipidomic profiles, the identified lipids can be connected to the atrial natriuretic factor *via* phosphatidylinositol- and phosphatidylcholine-dependent phospholipases C (62). Moreover, the natriuretic peptide-C receptor was also determined to induce the attenuation of adenylyl cyclase signaling, which activates the phosphatidylinositol turnover in vascular smooth muscle cells (63). However, further investigation is needed to clarify the effect of the TTS lipidomic profiles on the NT-proBNP/myoglobin and NT-proBNP/troponin T concentrations in order to develop novel lipid-dependent biomarker ratios for TTS.

## Limitations of Study

Our study revealed that lipidomic profiling in TTS patients was significantly different from controls. Further research is required to elucidate the significance of altered lipid compositions and quantity in acute TTS and subacute TTS in relation to ACS.

## Conclusion

Our study revealed a detailed overview of lipid classes and absolute quantitative information on the individual lipid species and their distribution pattern from the blood of TTS patients using high-throughput tandem mass spectrometry. Our investigation links lipid and inflammation biology; the computational pathway and network analyses draw attention to an intriguing diversity of targetable mechanisms potentially relevant to prevent primary or recurrent TTS in the future.

## Data Availability Statement

The original contributions presented in the study are included in the article/Supplementary Material, further inquiries can be directed to the corresponding authors.

## Ethics Statement

The studies involving human participants were reviewed and approved by Hiroshima City Asa Hospital Research Committee (01-3-3), Hiroshima, Japan. The patients/participants provided their written informed consent to participate in this study.

## Author Contributions

SK, RR, and DK: data analysis and visualization. SK, GG, RR, and CYF: writing–review and editing draft. SS and CYF: computational analyses. MH and GL: lipid analysis. MN and SE: sample collection and validation. SK: supervision. SK and CYF: designed and conducted. All authors read the study and approved the manuscript for publication.

## Funding

We are grateful to Stiftung Forschung hilft and DFG 515/5-1 for the research grant to CYF.

## Conflict of Interest

The authors declare that the research was conducted in the absence of any commercial or financial relationships that could be construed as a potential conflict of interest.

## Publisher's Note

All claims expressed in this article are solely those of the authors and do not necessarily represent those of their affiliated organizations, or those of the publisher, the editors and the reviewers. Any product that may be evaluated in this article, or claim that may be made by its manufacturer, is not guaranteed or endorsed by the publisher.
