# Supplementary material for: Quantitative Lipidomic Analysis of Takotsubo Syndrome Patients' Serum
Source: Front Cardiovasc Med. 2022 Apr 19;9:797154. doi: 10.3389/fcvm.2022.797154 (PMC9062978; doi:10.3389/fcvm.2022.797154)
Supplement: Supplementary file 1 [file Data_Sheet_1.docx]

Quantitative lipidomic analysis of Takotsubo syndrome patients’ serum

**Supplementary Material**


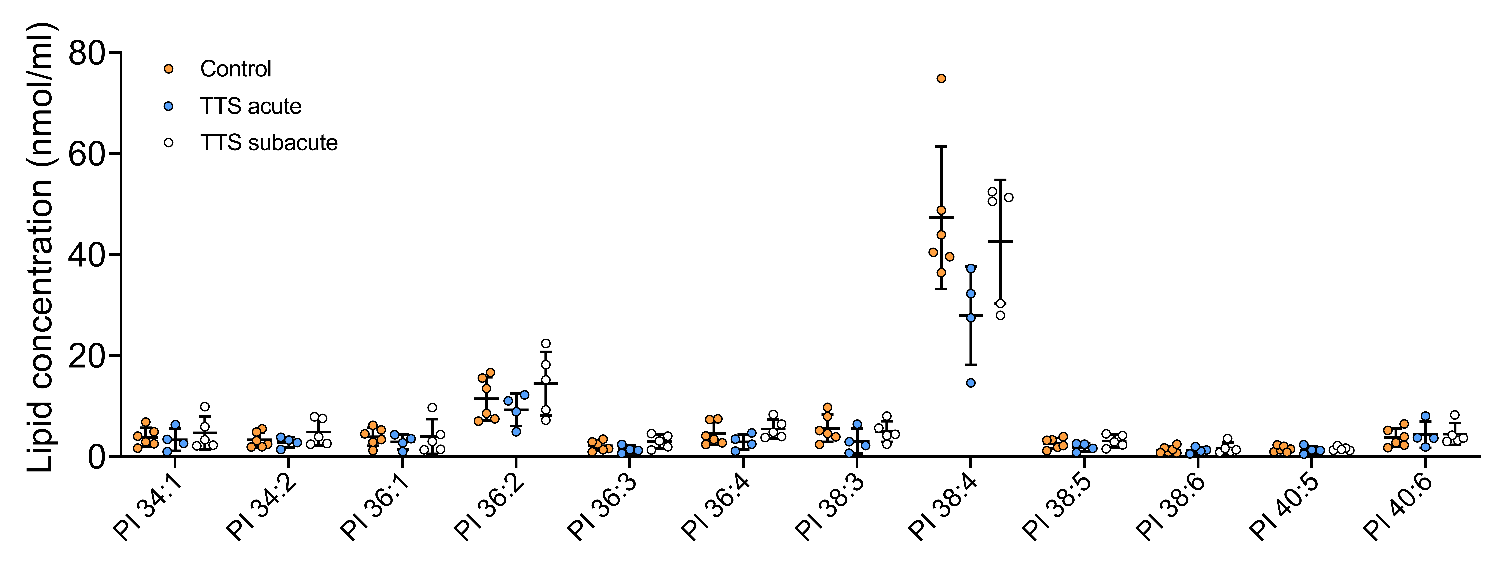


**Supplementary figure 1. Composition of individual PI lipid species in the acute TTS, subacute** **TTS, and control groups.** Values are represented as nmol/ml. Values are mean ± SD, where significance is not mentioned, values are considered as being not significant. PI: Phosphatidylinositol, TTS: Takotsubo syndrome.


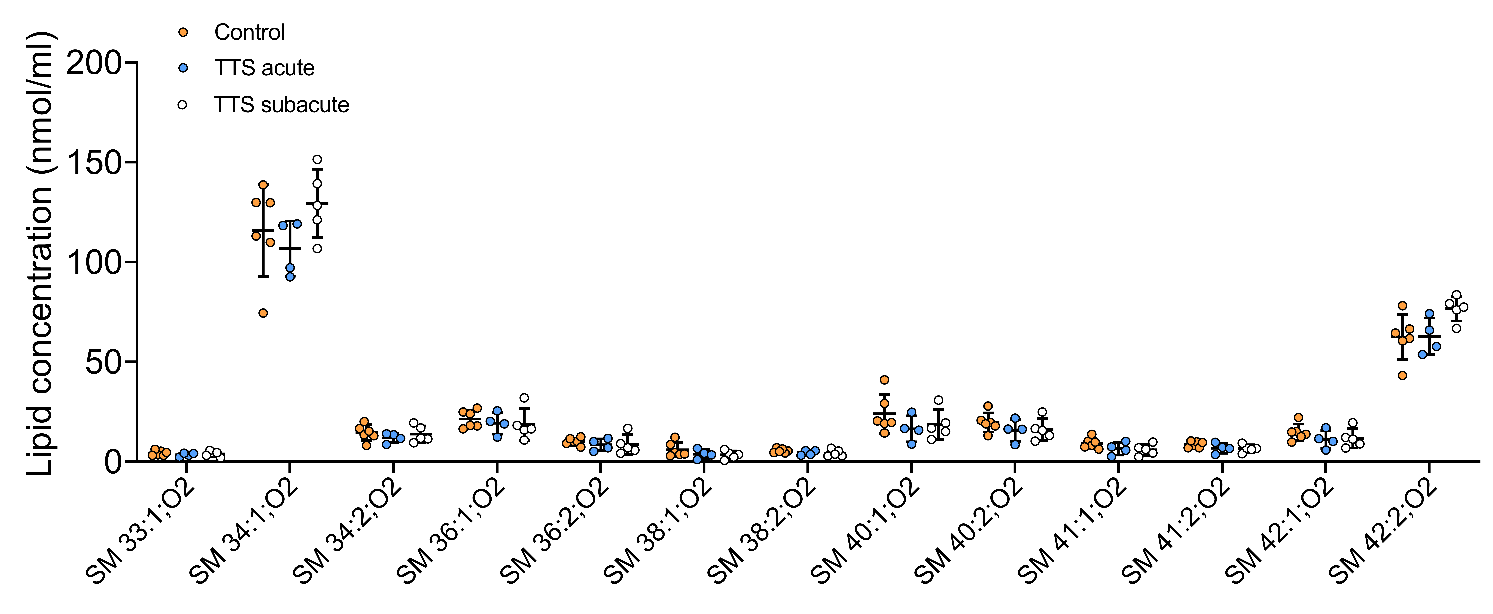


**Supplementary figure 2. Composition of individual SM lipid species in the acute TTS, subacute TTS, and control groups.** Values are represented as nmol/ml. Values are mean ± SD. Where significance is not mentioned, values are considered as being not significant. SM: Sphingomyelin, TTS: Takotsubo syndrome.


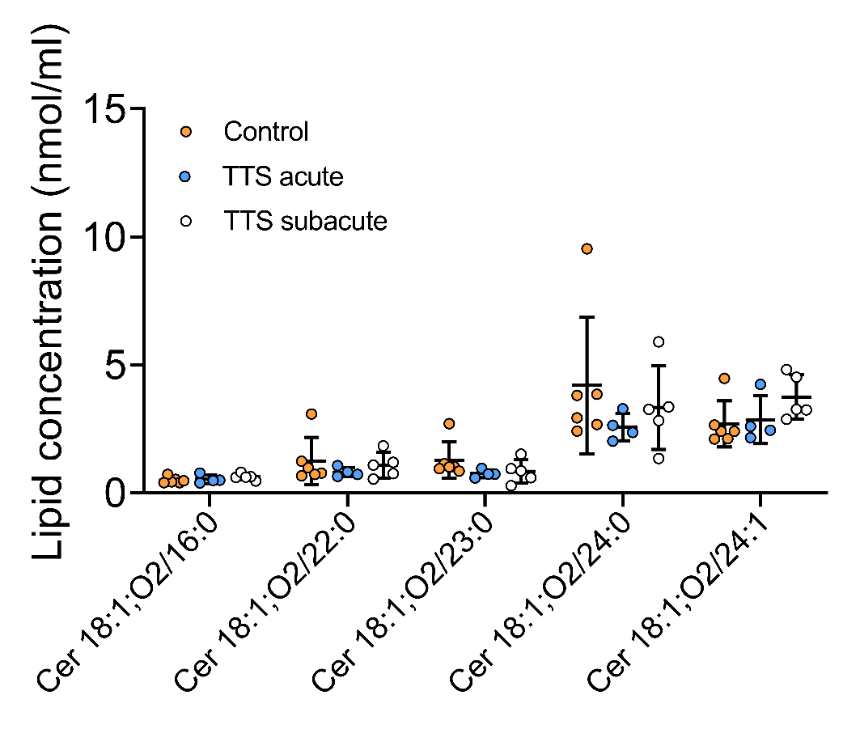


**Supplementary figure 3. Composition of individual Cer lipid species in the acute** **TTS, subacute TTS, and control groups.** Values are represented as nmol/ml. Values are mean ± SD. Where significance is not mentioned, values are considered as being not significant. Cer: Ceramide, TTS: Takotsubo syndrome.


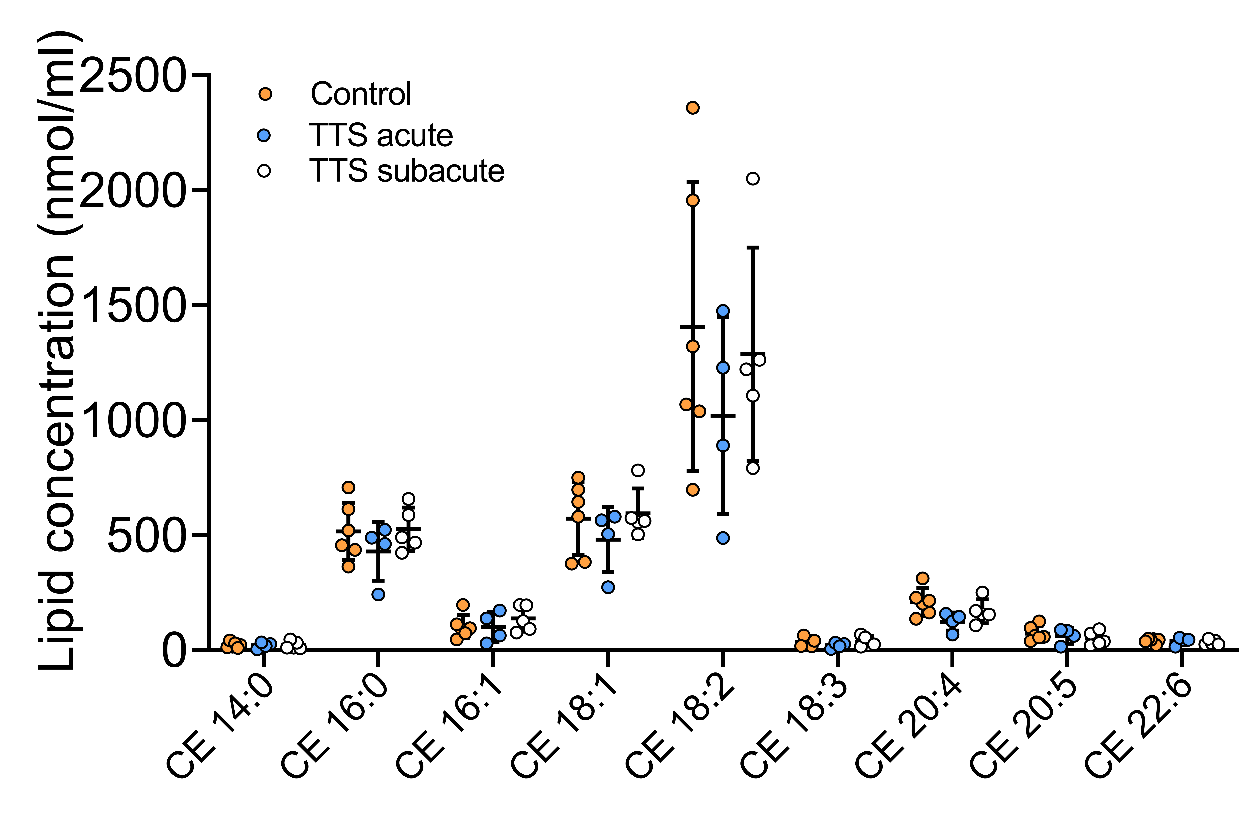


**Supplementary figure 4. Composition of individual CE lipid species in the acute TTS, subacute TTS, and control groups.** Values are represented as nmol/ml. Values are mean ± SD. Where significance is not mentioned, values are considered as being not significant. CE: Cholesteryl esters, TTS: Takotsubo syndrome.


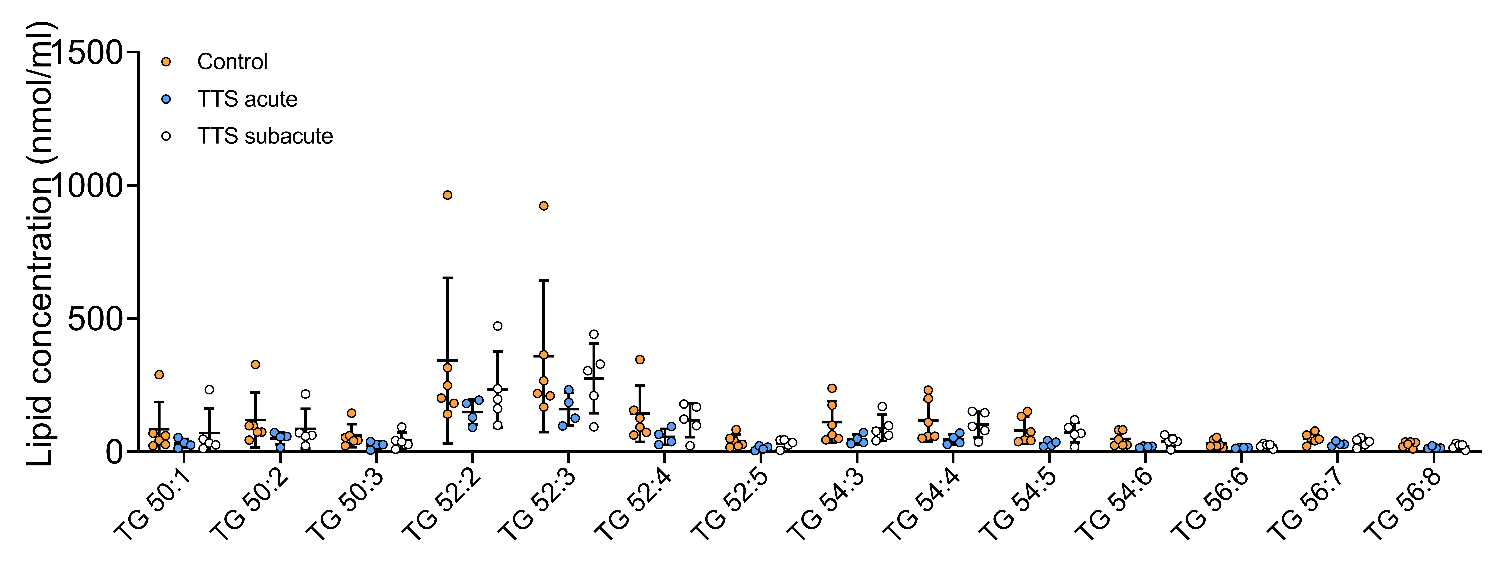


**Supplementary figure 5. Composition of individual TG lipid species in the acute TTS, subacute TTS, and control groups.** Values are represented as nmol/ml. Values are mean ± SD. Where significance is not mentioned, values are considered as being not significant. TG: Triacylglycerol, TTS: Takotsubo syndrome.
